# Significance of the p38MAPK-CRP2 axis in myofibroblastic phenotypic transition

**DOI:** 10.1247/csf.23060

**Published:** 2023-10-27

**Authors:** Ken’ichiro Hayashi, Reuben Jacob Labios, Tsuyoshi Morita, Atsushige Ashimori, Ren Aoki, Masanori Mikuni, Kazuhiro Kimura

**Affiliations:** 1 Department of RNA Biology and Neuroscience, Osaka University Graduate School of Medicine, 2-2 Yamadaoka, Suita, Osaka 565-0871, Japan; 2 Department of Biology, Wakayama Medical University School of Medicine, 580 Mikazura, Wakayama 641-0011, Japan; 3 Department of Ophthalmology, Yamaguchi University Graduate School of Medicine, Minami-Kogushi 1-1-1, Ube, Yamaguchi 755-8505, Japan

**Keywords:** CRP2, p38MAPK, MRTF, myofibroblasts, retinal pigment epithelial cells

## Abstract

We have recently demonstrated that a LIM domain protein, cysteine and glycine-rich protein 2 (CSRP2 [CRP2]), plays a vital role in the functional expression of myofibroblasts and cancer-associated fibroblasts. CRP2 binds directly to myocardin-related transcription factors (MRTF [MRTF-A or MRTF-B]) and serum response factor (SRF) to stabilize the MRTF/SRF/CArG-box complex, leading to the expression of smooth muscle cell (SMC) genes such as α-smooth muscle actin (α-SMA) and collagens. These are the marker genes for myofibroblasts. Here, we show that the adhesion of cultured human skin fibroblasts (HSFs) to collagen reduces the myofibroblastic features. HSF adhesion to collagen suppresses the expression of CRP2 and CSRP2-binding protein (CSRP2BP [CRP2BP]) and reduces the expression of SMC genes. Although CRP2BP is known as an epigenetic factor, we find that CRP2BP also acts as an adaptor protein to enhance the function of CRP2 mentioned above. This CRP2BP function does not depend on its histone acetyltransferase activity. We also addressed the molecular mechanism of the reduced myofibroblastic features of HSFs on collagen. HSF adhesion to collagen inhibits the p38MAPK-mediated pathway, and reducing the p38MAPK activity decreases the expression of CRP2 and SMC genes. Thus, the activation of p38MAPK is critical for the myofibroblastic features. We also show evidence that CRP2 plays a role in the myofibroblastic transition of retinal pigment epithelial cells (RPEs). Like HSFs, such phenotypic modulation of RPEs depends on the p38MAPK pathway.

## Introduction

Myofibroblasts are mesenchyme cells with smooth muscle cell (SMC)-like features and play a role in wound healing and tissue repair (Hinz and Lagaress, 2020). Myofibroblasts ectopically express SMC genes such as α-smooth muscle actin (α-SMA) and collagens. This ectopic expression is the hallmark of myofibroblasts. Several cells, fibroblasts, epithelial cells, circulating bone marrow-derived stem cells, and endothelial cells (ECs) are known as a source of myofibroblasts. The phenotypic transition from fibroblasts to myofibroblasts or trans-differentiation from epithelial cells to myofibroblasts is a potential source. This transition caused by inflammatory stress is known as an epithelial-mesenchymal transition (EMT) (Morita *et al.*, 2007; Karagiannis *et al.*, 2012). Under the inflammatory conditions, multipotential cytokines such as transforming growth factor-βs (TGF-βs) activate the transcription mediated through the myocardin-related transcription factors (MRTF-A and MRTF-B [MRTF])/SRF/CArG-box (Posern and Treisman, 2006; Gasparics and Sebe, 2018), leading to the promotion of the SMC gene expression. In general, activation of Rho signaling followed by nuclear accumulation of MRTF triggers the onset of EMT (Morita *et al.*, 2007; Posern and Treisman, 2006; Gasparics and Sebe, 2018). We have recently found that a LIM domain protein, cysteine and glycine-rich protein 2 (CSRP2 [CRP2]), plays a vital role in the transcription of SMC genes; CRP2 binds directly to MRTF and SRF to stabilize the MRTF/SRF/CArG-box complex in TGF-β2-stimulated human skin fibroblasts (HSFs), leading to the activation of SMC gene expression. Since TGF-β induces the expression of CRP2, which is closely related to SMC gene expression, we conclude that CRP2 is a vital factor for the myofibroblastic features (Hayashi *et al.*, 2023).

Here, we have clarified the relationship among CRP2 and SMC gene expression, adhesion to collagen, and signaling pathways mediated by p38MAPK in HSF. Although a CSRP2-binding protein (CSRP2BP [CRP2BP]) is known as an epigenetic factor, we find that CRP2BP also acts as an adaptor protein to enhance the function of CRP2 mentioned above. We further show the possibility that CRP2 contributes to the myofibroblastic transition of retinal pigment epithelial cells (RPEs). This phenotypic transition is the cause of retinal fibrosis occurring in developing neovascular age-related macular degeneration (nAMD), suggesting the significance of CRP2 in the development of retinal degeneration.

## Materials and Methods

### Reagents and antibodies

SB203580 was purchased from Caymen Chemical. TGF-β2 and TGF-β1 were purchased from PeproTec. Antibodies used in this study are as follows: anti-CRP2 (CSRP2) [HPA045617], anti-MRTF-B (MKL2) [HPA001286], anti-α-SMA [A2547], CRP2BP [SAB2700111], and anti-α-tubulin [T9026] antibodies, and anti-FLAG M2 gel (Sigma); anti-HA affinity matrix and anti-HA (3F10) antibody (Roche Applied Science); anti-DYKDDDDK (anti-Flag) antibody (Trans Genic, KO602-M); anti-SRF [#5147], anti-P-p38MAPK [#9211] antibodies (Cell Signaling Technology); anti-glyceraldehyde-3-phosphate dehydrogenase (GAPDH) antibody (Ambion, AM4300); anti-MRTF-A [sc390324], anti-p38MAPK [sc535] antibodies (Santa Cruz Biotechnology). Secondary antibodies were conjugated to Alexa Flour 568 or Alexa Flour 488 (Molecular Probes). Type I collagen (Cellmatrix Type I-A) was purchased from Nitta Gelatin Inc.

## Plasmids and siRNAs

Construction of the expression plasmids for Flag-tagged mouse MRTF-A, Myc-tagged human SRF (wild-type and mutants), and HA-tagged human CRP2 was previously described (Hayashi and Morita, 2013; Hayashi *et al.*, 2023). The cDNA of human CRP2BP (NCBI Reference Sequence: NM_020536) with the indicated tag was amplified by RT-PCR and inserted into an expression plasmid, pCS2+. The sequences of newly constructed plasmids were confirmed. The following siRNAs (Sigma) were used: CRP2 (Hs_CSRP2_1147 and SASI_Hs02_00331621) and a scrambled siRNA (control siRNA).

### Cell culture and transfection

Primary-cultured normal HSFs (CRL-2072) and human telomerase reverse transcriptase-immortalized retinal pigment epithelial (hTERT-RPE1) cells (CRL-400) was purchased from ATCC. They were cultured in Dulbecco’s modified Eagle’s medium (DMEM) supplemented with 10% fetal calf serum for HSFs and DMEM/F-12 (1:1) supplemented with 10% fetal calf serum for hTERT-RPE1 cells, respectively. HSFs were cultured under the indicated conditions, non-coated dishes [none], COLI thin film (COLI-film, or COLI-gel. hTERT-RPE1 cells were cultured on non-coated dishes. COLI thin films and COLI gels were prepared according to the manufacturer’s instructions. We used ViaFect (Promega) or Lipofectamine RNAiMAX (Invitrogen) for transfection.

### RT-qPCR

We used PrimeScript 1st strand cDNA Synthesis Kit (Takara Bio). RT-qPCR was performed using the Brilliant III Ultra-Fast SYBR QPCR MasterMix (Agilent Technologies) and a LightCycler Nano (Roche Life Science). The expression of each mRNA was normalized to GAPDH mRNA expression. This analysis was performed in triplicate and repeated three times. Their levels in control cells were set at 100% (means ± SEMs of the results from at least three independent experiments). The specific primers used in this study are as follows: GAPDH sense primer, ACTCCTCCACCTTTGACGCTG; GAPDH antisense primer, GCCAAATTCGTTGTCATACCAGGAA; α-SMA sense primer, AATCCTGTGAAGCAGCTCCAG; α-SMA antisense primer, CCCCTGATGTCTGGGACGT; COLI a1 sense primer, AAGAATGGAGATGATGGGGAAG; COLI a1 antisense primer, CTTAGGACCAGCAGGACCAG; CRP2 sense primer, AGCCCAGCCTCGCTAGCTC; CRP2 antisense primer, CAGAGAAAAGCAGCAGCGGTG; CRP1 sense primer, AATGCCGAACTGGGGAGGAG; CRP1 antisense primer, CACAGTGGTACTGTCCAGATTC; TGF-β2 sense primer, GCCCTCCTACAGACTGGAGTCA; TGF-β2 antisense primer, GAAGGCAGCAATTATCCTGCAC; TGF-β1 sense primer, TGGAAGTGGATCCACGAGC; TGF-β1 antisense primer, TCAGCTGCACTTGCAGGAG; TGF-βR1 sense primer, GTTTGTATGTGCACCCTCTTC; TGF-βR1 antisense primer, ACTGGTCCAGCAATGACAGC; TGF-βR2 sense primer, GGCCGCTGCACATCGTCC; TGF-βR2 antisense primer, GTTGTGGAAACTTGACTGCAC; TGF-βR3 sense primer, TGGACGAGACGCACTGTTGG; TGF-βR3 antisense primer, CAGGATGGGAGGCACTGAC; Thymosin β4 sense primer, CCTCCGCAACCATGTCTGA; Thymosin β4 antisense primer, ATGCTCGTGGAATGTACAGTGC; CRP2BP sense primer, TTCAGGTGGAAAGAAGATATCTG; CRP2BP antisense primer, TTCCACCATCCTGGCTCTCC; SRF sense primer, CAGCTTCACCCTCATGCCTG; SRF antisense primer, ATGGTGGCGGGCAGCGTC; MRTF-A sense primer, GTCAGGATGCACATTTTGGAAG; MRTF-A antisense primer, TTTGGGATAGTTCACCTGGCC; MRTF-B sense primer, ACCGAGGATGAAGTGGGACC; MRTF-B antisense primer, GTTGCAGCCTCAGCTGGAGC.

### Immunocytochemistry

Fluorescent images were collected using a Biorevo BZ-X700 fluorescence microscope (Keyence). Subcellular localization of MRTF was classified into three groups: nuclear-specific localization (N); diffuse distribution in the nucleus and the cytoplasm (NC); and cytoplasmic localization (C) (Hayashi and Morita, 2013; Hayashi *et al.*, 2015). Classification of MRTF subcellular localization depends on nuclear/cytoplasmic (N/C) signal intensity, and the criteria are as follows: N/C >75%, nuclear-specific localization; N/C = 50~75%, diffuse distribution in the nucleus and the cytoplasm; N/C <50%, cytoplasmic localization. For each experiment (at least three independent experiments), 100–200 cells were analyzed. The proportions of cells that exhibited the respective expression patterns were determined (means ± SEMs). The fluorescence intensities were quantified using NIH ImageJ software (ROI Manager).

### IB analysis

Protein samples were separated by SDS-PAGE and then transferred to poly vinylidene fluoride (PVDF) membranes (Immobilon-P) (Merck Millipore). IB signals were detected using Immuno-Star (FUJIFILM Wako Pure Chemical Corporation) and C-DiGit Blot Scanner (LI-COR Biosciences). α-tubulin and/or GAPDH were used as loading controls. Molecular mass markers (kDa) are on the side of IB data. This analysis was repeated at least three times. Quantification of each IB signal intensity was performed using NIH ImageJ software. The blot membranes were cut into several pieces and incubated with the specified antibodies. Concerning the IB with anti-P-p38MAPK antibody and anti-p38MAPK antibody, the same membranes were first incubated with anti-P-p38MAPK antibody and then incubated with p38MAPK antibody after stripping the anti-P-p38MAPK antibody.

### Promoter assay

Cultured cells were transfected with a 3xCArG-box-Luciferase reporter plasmid (Morita *et al.*, 2007) (500 ng), pSV-β-gal (300 ng), and the indicated expression plasmids (100 ng) and then cultured for 48 hours. Luciferase and β-galactosidase activities were measured by Luciferase Assay Kit (Promega) and β-galactosidase Assay Kit (Takara Bio). Relative luciferase activity was expressed in luminescence units normalized to the β-galactosidase activity of pSVβ-gal. Promoter activity in the control cells indicated was set at 100% (means ± SEMs). These assays were performed in triplicate and repeated at least twice.

### Protein-protein interaction analysis

Protein-protein interaction assay was performed as described elsewhere (Hayashi and Morita, 2013; Hayashi *et al.*, 2014). The indicated proteins were prepared using a TNT SP6 high-yield expression system based on an optimized wheat germ extract (Promega). The IP mixtures (500 μl) containing the indicated in vitro translated proteins were subjected to IP/IB analysis with the indicated antibodies. These analyses were repeated three times. The respective IP/IB signal intensities were quantified using NIH ImageJ software. Relative binding affinity was calculated as follows: the ratio of coimmunoprecipitated protein signal intensity to immunoprecipitated protein signal intensity was normalized by the value of the control experiment, which was set at 100% (mean ± SEMs).

### DNA affinity binding assay

Biotinylated 3xCArG-box probes (Morita *et al.*, 2007) were incubated with Streptavidin M-280 Dynabeads (Invitrogen) according to the manufacturer’s instructions. The detailed procedures were described in our previous study (Hayashi *et al.*, 2015). In brief, the Dynabeads-3xCArG-box probe was mixed with the indicated in vitro translated proteins and incubated with rotation for 3 hours at 4°C. After washing, the probe-bound proteins were subjected to IB analysis with the specified antibodies. Quantification of each IB signal intensity was performed using NIH ImageJ software. Binding affinity in the indicated experiments was set to 100% (mean ± SEM of results from at least three independent experiments).

### Wound healing assay

Indicated cells were grown to 70–80% confluency and were transfected with the indicated siRNAs. The cells were further cultured for 2 days. In the case of TGF-β2 stimulation, cells were cultured in low serum (0.3%) medium after siRNA transfection and then stimulated with TGF-β2 (5 ng/ml). Confluent cells were scratch-wounded with a 20 μl pipette tip. Cell migration was monitored using a low-light inverted Olympus microscope (CKX 41) at 0 and 16 hours after scratching. Consecutive areas (4–5 images) were analyzed with NIH ImageJ software to quantify the migration areas. The details are as follows: we traced the area that was not cell-migrated (immediately after scratching and after 16 hours) with Freehand Line and measured it to calculate the cell migration area. Percentages indicate the relative migration areas normalized by the migration areas of control cells, which were set at 100% (means ± SEMs of the results from five serial places). These assays were repeated three times.

### Statistical analysis

Results are presented as means and standard errors. We statistically analyzed the data using a two-tailed paired student t-test and used an ANOVA with Bonferroni correction for the multi-data. The significance level is set at 0.05. P-values indicate the statistical significance using asterisks (non-specific [ns]: P>0.05; *: P<0.05; **: P<0.01; ***: P<0.001: ****: P<0.0001).

## Results and Discussion

### Decreased myofibroblastic phenotype of HSFs by attachment to type I collagen (COLI)

**Fig. 1:**
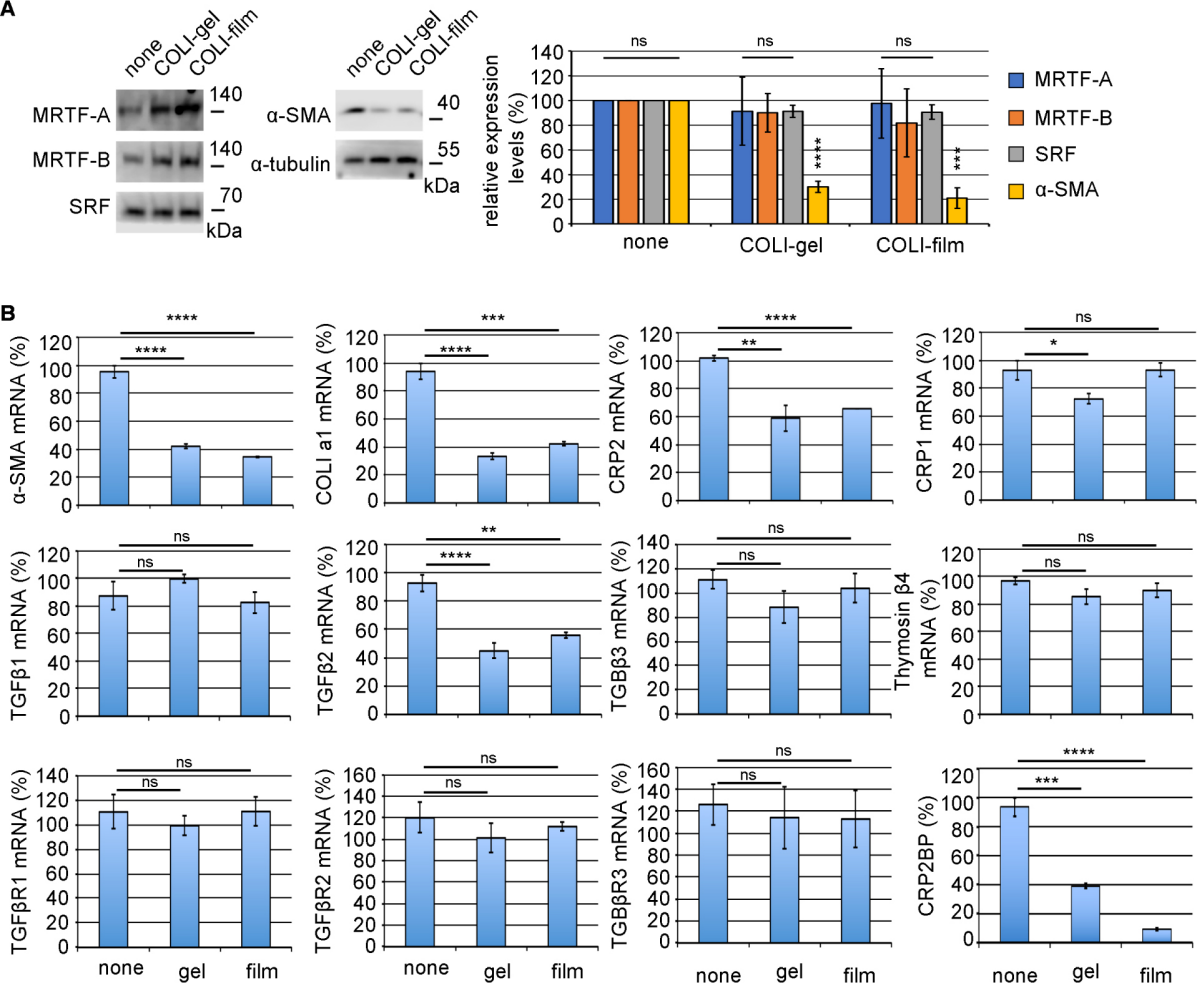
Decreased myofibroblastic phenotype of HSFs by attachment to type I collagen (COLI) (A) HSFs were cultured on non-coated dishes (none), COLI thin film (COLI-film), or COLI-gel for 4 days. Whole-cell lysates were subjected to IB (left panel). The right graph shows the relative expression levels of each protein, MRTF-A, MRTF-B, SRF, and α-SMA. Their levels in HSFs cultured on non-coated dishes (none) were set at 100% (means ± SEMs of the results from multiple independent experiments, n = 3). Only the α-SMA expression significantly reduces in HSFs cultured on COLI-gel or COLI-film. ANOVA shows a significant difference in the expression levels of α-SMA among the three culture conditions (P<0.0001). Asterisks indicate P-values for multiple comparisons of the expression of α-SMA protein (pair to HSFs on non-coated dishes and HSFs on COLI-gel or COLI-film). (B) The expression of myofibroblast markers and the related factors. HSFs were cultured under the indicated conditions: non-coated dish (none), COLI-gel (gel), and COLI-film (film). RT-qPCR quantified the expression levels of the indicated mRNAs. Their levels in HSFs cultured on non-coated dishes were set at 100% (means ± SEMs of the results from multiple independent experiments, n = 3). ANOVA shows no significant difference in the expression levels of TGF-β1, TGF-β3, thymosin β4, and TGF-βR1-3 among the three culture conditions (P>0.05). Asterisks indicate the statistical significance of multiple comparisons between the indicated pairs (ns: P>0.05; *: P<0.05; **: P<0.01; ***: P<0.001: ****: P<0.0001).

A function of fibroblasts is the production of ECM, whose ability is weak under quiescent conditions. However, myofibroblasts transited from fibroblasts potently synthesize ECM to heal injured tissues under inflammatory stress conditions. We speculate that HSF attachment to ECM may maintain the quiescent phenotype. To test this hypothesis, we examined the effect of collagen on the phenotype of primary-culture HSFs that exhibit myofibroblastic phenotype (α-SMA-positive) under the usual culture conditions (culture on non-coated dishes) (Hayashi *et al.*, 2023). As myofibroblast differentiation closely correlates with MRTF, SRF, and α-SMA, we compared their expression in HSFs cultured on non-coated dishes, COLI thin film (COLI-film), or COLI-gel. The α-SMA expression decreases in HSFs on both collagens. However, the expression levels of MRTF and SRF are not affected (Fig. 1A). We then analyzed the expression of SMC genes and the related transcription factors focusing on the SRF-mediated transcription by real-time quantitative PCR (RT-qPCR) (Fig. 1B). The expression levels of α-SMA, COLI a1, CRP2, CRP2BP, and TGF-β2 mRNAs are significantly low in HSFs on both collagens. However, the suppressive effects of collagens on the expression of TGF-β1, TGF-β3, thymosin β4, three types of TGF-β receptors, and CRP1 are less significant or weak. The culture conditions do not affect the subcellular localization of MRTF (Fig. S1). These findings suggest that HSF attachment to collagens suppresses the expression of SMC genes even though MRTF is in the nucleus. Nuclear accumulation of MRTF does not depend on actin dynamics (Hayashi *et al.*, 2023). However, the question of why MRTF localize constitutively to the nucleus and why actin dynamics do not affect their localization remains unanswered. The expression balance of importins and exportins and the binding affinity of MRTF to SRF, which play a significant role in the nuclear localization of MRTF, may regulate the subcellular localization of MRTF (Hayashi and Morita, 2013). We recently demonstrated that CRP2 plays a significant role in SMC gene expression in HSFs (Hayashi *et al.*, 2023). The basis of this finding is as follows: CRP2 binds directly to MRTF and serum response factor (SRF) to stabilize the MRTF/SRF/CArG-box complex leading to the expression of smooth muscle cell (SMC) genes, and the knock-down (KD) of CRP2 reduces the SMC gene in HSFs cultured on non-coated dishes. Thus, we speculate that the down-regulation of CRP2 is the primary reason for the repression of SMC gene expression in HSF on collagen. Interestingly, the expression of CRP2BP coincides with the SMC gene and CRP2 expression. Since the SRF/CRP2/GATA-mediated transcription requires CRP2BP in VSMCs (Ma *et al.*, 2017), we speculate cooperative transcriptional regulation of CRP2 and CRPBP in the expression of SMC genes. In this case, CRP2BP act as an epigenetic factor, histone acetyltransferase (HAT), to activate the SRF-mediated transcription. Here, we further investigated the role of CRP2BP in the CRP2-mediated MRTF/SRF/CArG-box-dependent transcription in myofibroblasts.

### Role of CRP2BP other than epigenetic function in the transcriptional regulation via MRTF/SRF/CArG-box

**Fig. 2:**
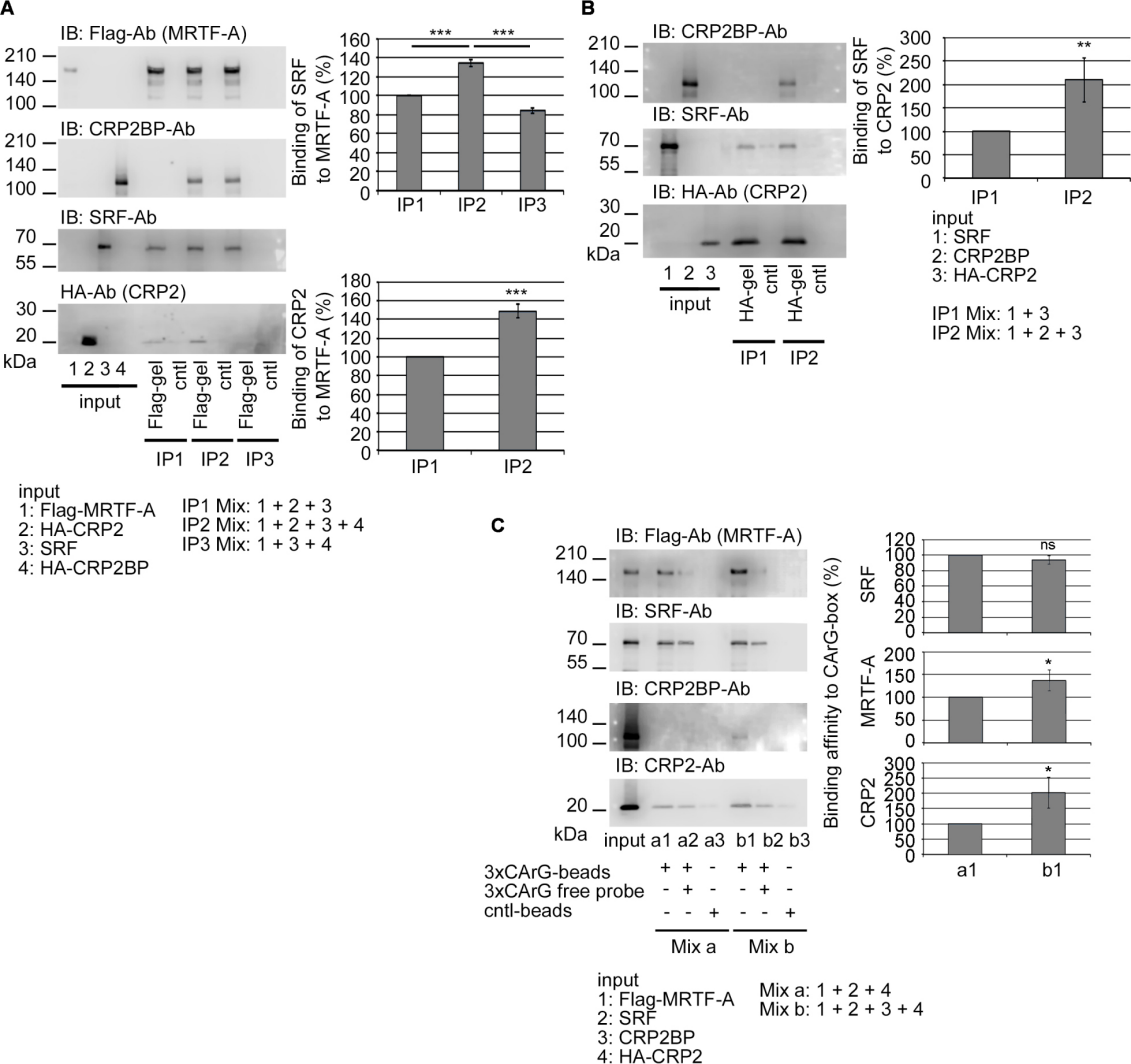
Effect of CRP2BP on the recruitment of MRTF-A/SRF/CRP2 to the CArG-box (A and B) Protein-protein interaction analysis using in vitro translated proteins. Mixtures of the indicated proteins (numbering proteins in the input panels) were subjected to IP/IB analysis as described in Materials and Methods. Interactions among MRTF-A, SRF, and CRP2 or CRP2BP, or these four proteins (A). The relative binding affinity of SRF to MRTF-A (right upper graph, A) and that of CRP2 to MRTF-A (right lower graph, A). The affinity levels in the IP2 and IP3 Mixes were normalized using the affinity level in the IP1 Mix as 100% (means ± SEMs of the results from multiple independent experiments, n = 3). ANOVA shows a significant difference in the binding affinity of SRF to MRTF-A among the three Mixes (IP1-IP3) (P<0.0001). Effect of CRP2BP on the interaction between SRF and CRP2 (B). The relative binding affinity of SRF to CRP2 (right graph, B). The affinity level in the IP2 Mix was normalized as described above. Quantified results are means ± SEMs of the results from multiple independent experiments (n = 3). (C) DNA affinity binding assay using in vitro translated proteins. Mixtures of the marked proteins (Mix a and Mix b) were pulled down with 3xCArG-box-Dynabeads (a1, a2, b1, b2) or control Dynabeads (cntl-beads) (a3, b3) in the absence or presence of free 3xCArG-box probes. IB shows the 3xCArG-box-bound proteins (left panel). The graphs show the quantification of the binding affinity of each protein to the 3xCArG-box-Dynabeads. In each experiment, the binding affinity in the absence of CRP2BP (a1) was set at 100% (means ± SEMs of the results from multiple independent experiments, n = 3). Asterisks indicate the statistical significance of multiple comparisons between the indicated pairs (ns: P>0.05; *: P<0.05; **: P<0.01; ***: P<0.001).

**Fig. 3:**
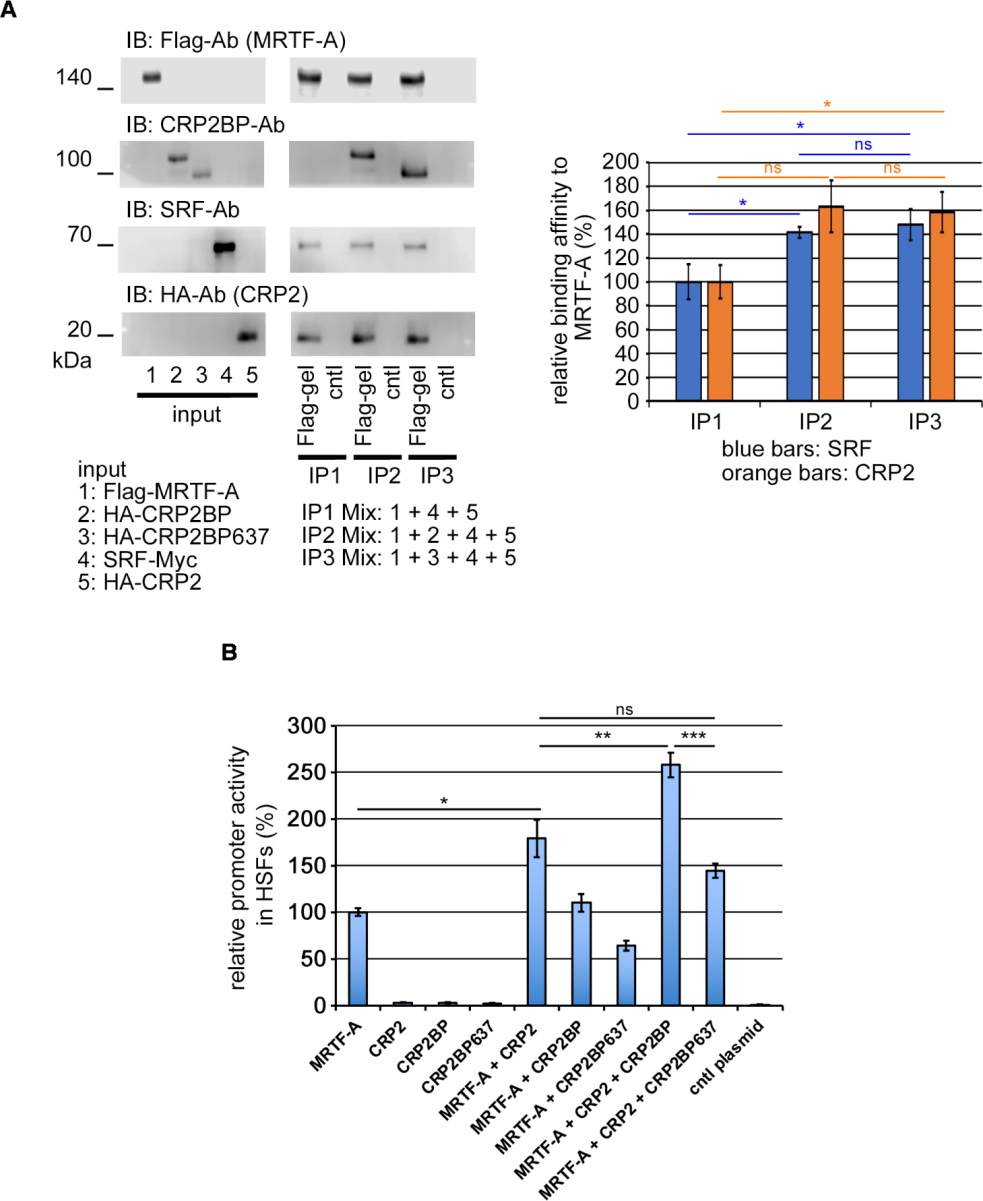
Effect of CRP2BP on the transcriptional activity mediated by the CArG-box (A) Protein-protein interaction analysis using in vitro translated proteins. Mixtures of the indicated proteins (numbering proteins in the input panels) were subjected to IP/IB analysis as described in Materials and Methods. Interactions among MRTF-A, SRF, CRP2 and CRP2BP or CRP2BP637 were examined as described in the legend of Fig. 2A, B. The graph shows the relative binding affinity of SRF or CRP2 to MRTF-A. In each experiment, the respective affinity levels in the IP2 and IP3 Mixes were normalized using the affinity level in the IP1 Mix as 100% (means ± SEMs of the results from multiple independent experiments, n = 3). ANOVA shows a significant difference in the affinity levels of SRF and CRP2 to MRTF-A among the three binding conditions (P = 0.0122 for SRF and P = 0.0214 for CRP2). (B) Assessment of synergistic effects of MRTF-A, CRP2, and CRP2BP or CRP2BP637 on the CArG-box-dependent promoter activity in HSFs on non-coated dishes. Cells were transfected with 3xCArG-box-Luciferase reporter plasmid, pSVβ-gal, and the indicated plasmids or control plasmid. The promoter activity induced by exogenous MRTF-A alone was set at 100% (means ± SEMs of the results from multiple independent experiments, n = 3). ANOVA shows a significant difference in promoter activity among the ten different assays (P<0.0001). Asterisks indicate the statistical significance of multiple comparisons between the indicated pairs (ns: P>0.05; *: P<0.05; **: P<0.01; ***: P<0.001).

Since CRP2 acts as an adaptor protein to stabilize the MRTF/SRF/CArG-box complex and synergistically activates the MRTF/SRF/CArG-box-dependent transcription (Hayashi *et al.*, 2023), we first examined the effect of CRP2BP on the stabilization of the MRTF/SRF/CArG-box complex by CRP2. Although CRP2BP further stabilizes the complex composed of SRF, MRTF-A, and CRP2 in vitro binding assays (Fig. 2A, B) and increases the binding affinity of MRTF-A and CRP2 to the CArG-box, CRP2BP does not affect SRF binding to the CArG-box (Fig. 2C). The C-terminal region of CRP2BP (amino acids 638–782) functions as a HAT and other domains contribute to binding to SRF and CRP2 (Ma *et al.*, 2017). In vitro binding assay reveals that CRP2BP and a mutant CRP2BP that lacks the HAT domain (CRP2BP637) similarly form the complex consisting of MRTF-A, SRF, and CRP2 (Fig. 3A). We then performed the promoter assay in HSFs on non-coated dishes to examine the role of the HAT domain of CRP2BP in the synergistic activation of MRTF/SRF/CArG-box-dependent transcription by CRP2 (Fig. 3B). Although cells expressing MRTF-A and CRP2 show the enhancement of MRTF-A/CArG-box-dependent promoter activity, cells expressing MRTF-A and CRP2BP do not cause such marked increasing activity. Co-expression of MRTF-A, CRP2, and CRP2BP further increases promoter activity, while CRP2BP637 does not cause such synergistic activation. These findings suggest that although the HAT domain of CRP2BP is critical for promoter activation, the N-terminal region of CRP2BP also acts as an adaptor protein to support the transcription regulated by MRTF-A/SRF/CRP2. However, the role of CRP2BP in MRTF/SRF/CArG-box-dependent transcription is ancillary to enhancing the function of CRP2 because co-expression of MRTF-A and CRP2BP does not cause a marked increase in promoter activity and TGF-β2 stimulation does not induce CRP2BP expression in HSFs (Hayashi *et al.*, 2023). This report is the first demonstration of the additional role of CRP2BP as an adaptor protein.

### Significance of the p38MAPK pathway in the myofibroblastic phenotype of HSFs

**Fig. 4:**
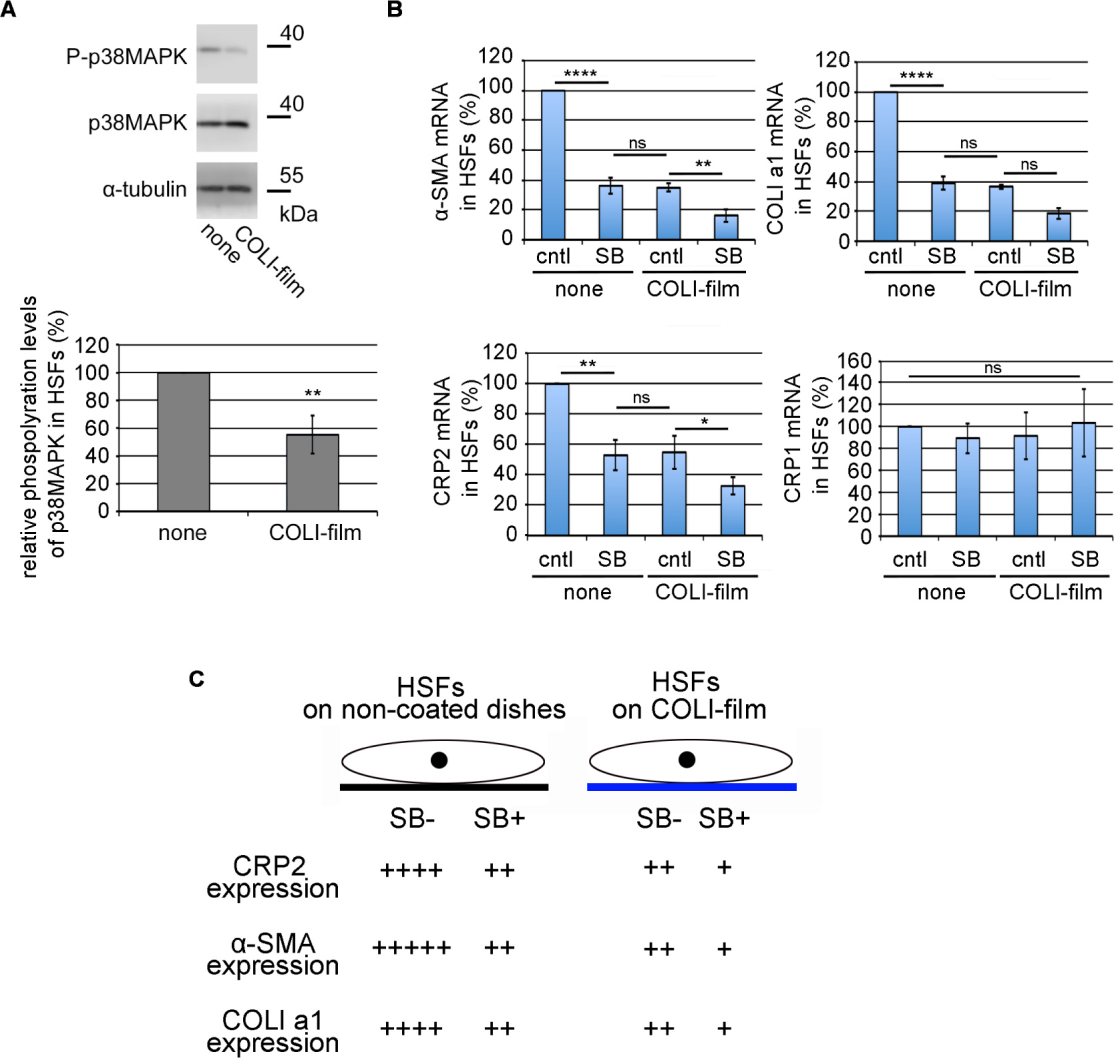
Roles of the p38MAPK pathway for the myofibroblastic phenotype of HSFs (A) Suppression of p38MAPK activity by HSF attachment to COLI-film. IB analysis with whole-cell lysates from the indicated HSF cultured on non-coated dishes (none) or COLI-film (upper panel). The lower graph shows the phosphorylation ratios of p38MAPK (P-p38MAPK/p38MAPK) in the respective HSF cultures; the phosphorylation ratio in HSFs cultured on non-coated dishes was set at 100% (means ± SEMs of the results from multiple independent experiments (n = 3). (B) Effect of the p38MAPK inhibitor on the expression of SMC genes and CRPs 1 and 2. HSFs were cultured on non-coated dishes (none) or COLI-film in the presence of either vehicle (DMSO) (control [cntl]) or 10 μM SB20350 (SB) for 1 day. RT-qPCR quantified the expression levels of the indicated mRNAs. Their levels in HSFs treated with vehicles on non-coated dishes were set at 100% (means ± SEMs of the results from multiple independent experiments, n = 3). ANOVA shows a significant difference in the expression levels of α-SMA, COLI a1, and CRP2 (P<0.0001 for α-SMA and COLI a1, P = 0.0001 for CRP2) but not the expression levels of CRP1 (P = 0.9667) among the three culture conditions. Asterisks indicate the statistical significance of multiple comparisons between the indicated pairs (ns: P>0.05; *: P<0.05; **: P<0.01; ****: P<0.0001). (C) Schematic summary of the relationship between the p38MAPK pathway and SMC gene expression in HSFs cultured on non-coated dishes or COLI-film. HSF attachment to COLI-film decreases the activation of the p38MAPK pathway, which is necessary to activate the expression of CRP2 and its downstream SMC genes. + ~ +++++ indicate the relative activation levels of p38MAPK and the expression levels of α-SMA and COLI a1.

To address the relationship between the signaling pathway and HSF adhesion to COLI-film, we focused on the p38MAPK because this pathway is vital for the SRF-mediated transcription in vascular smooth muscle cells (VSMCs) (Martin-Garrido *et al.*, 2011). The phosphorylation level of p38MAPK under the non-stimulated conditions is lower in HSFs on COLI films compared to HSFs on non-coated dishes (Fig. 4A), suggesting the suppression of p38MAPK in HSFs on COLI-film. We examined the effect of the p38MAPK inhibitor (SB203580) on the expression of α-SMA, COLI a1, CRP2, and CRP1 mRNAs in HSFs. Inhibition of the p38MAPK pathway reduces the expression of α-SMA, COLI a1, and CRP2 but not CRP1 in HSFs cultured on non-coated dishes or COLI-film. We detect no significant differences in expression levels of α-SMA, COLI a1, and CRP2 between SB203580-treated HSFs on non-coated dishes and vehicle-treated (control) HSFs on COLI-film (Fig. 4B). Fig. 4C shows the schematic summary of these results. Taking together these results and our recent findings that CRP2 plays a vital role in the functional expression of myofibroblasts (Hayashi *et al.*, 2023), we conclude that the expression of these SMC genes is closely related to the p38MAPK pathway and the expression of CRP2.

To address the effect of phosphorylation of SRF by p38MAPK (Martin-Garrido *et al.*, 2011) on the binding affinity to MRTF-A or CRP2, we prepared two types of mutant Myc-tagged SRF protein (S103D and S103A). The key p38MAPK phosphorylation site, serine-103, is converted to aspartate (S103D, pseudo-phosphorylation mutant) or alanine (S103A, non-phosphorylation mutant). These mutations do not affect the binding affinity to MRTF-A or CRP2, suggesting that the p38MAPK pathway does not affect the MRTF-A/SRF/CRP2 complex formation (Fig. S2A). The promoter assay in HSFs shows comparable results with the binding assays. Forced expression of each SRF expression plasmid (wild-type, D103A mutant, or D103D mutant) similarly induces the luciferase activity (Fig. S2B). Thus, the down-regulation of SMC genes in HSFs cultured on COLI-film seems to be due to the suppression of CRP2 expression but not the modification of SRF by p38MAPK.

Although we have not yet elucidated this COLI property, signal transduction mediated by HSF attachment to COLI may play a role. The examples are as follows: activation of p38MAPK caused by ECM detachment induces anoikis, and the oxidative stress caused by mitochondrial dysfunction may relate to the activation of p38MAPK in HSFs detached from COLI (Owens *et al.*, 2009; Mason and Schafer, 2019). Radnaa *et al.* recently reported that the motility of cells with p38MAPK knock-out (KO) significantly decreases and that p38MAPK KO suppresses TGF-β-induced EMT (Radnaa *et al.*, 2022). This finding supports the results shown in Fig. 4. A transcriptional co-activator with a PDZ-binding motif (TAZ) is a significant factor in myofibroblastic phenotype transition (Speight *et al.*, 2016). The up-regulation of TAZ by p38MAPK-induced MRTF activation is a possible pathway for the activation of myofibroblasts (Miranda *et al.*, 2017). Thus, the down-regulation of TAZ is one of the other possibilities than the CRP2 down-regulation caused by reduced p38MAPK activity. The elastic moduli of COLI-gel and COLI-film are different, and such difference is closely related to cell properties (Fassett *et al.*, 2006). Although the mechanical properties regulate the p38MAPK activity, we speculate that cell adhesion to COLI rather than the elastic modulus would be significant because both collagens (COLI-gel and COLI-film) similarly suppress the myofibroblastic phenotype (Fig. 1). Further validation is necessary to elucidate this molecular mechanism. TGF-β-induced up-regulation of CRP2 depends on transcription factor AFT2 activation in VSMCs (Wu *et al.*, 2014). In this case, JNK, but not p38MAPK, plays a role in the activation of ATF2. In contrast, the activation of ATF2 requires the activation of p38MAPK in HEK293 cells and C2C12 myoblasts (Zhang and Li, 2012). In HSFs, the activation of ATF2 may depend on the p38MAPK pathway because inhibition of p38MAPK decreases the expression of CRP2 (Fig. 4B).

### Involvement of CRP2 in TGF-β2-induced myofibroblastic transition of hTERT-RPE1 cells

**Fig. 5:**
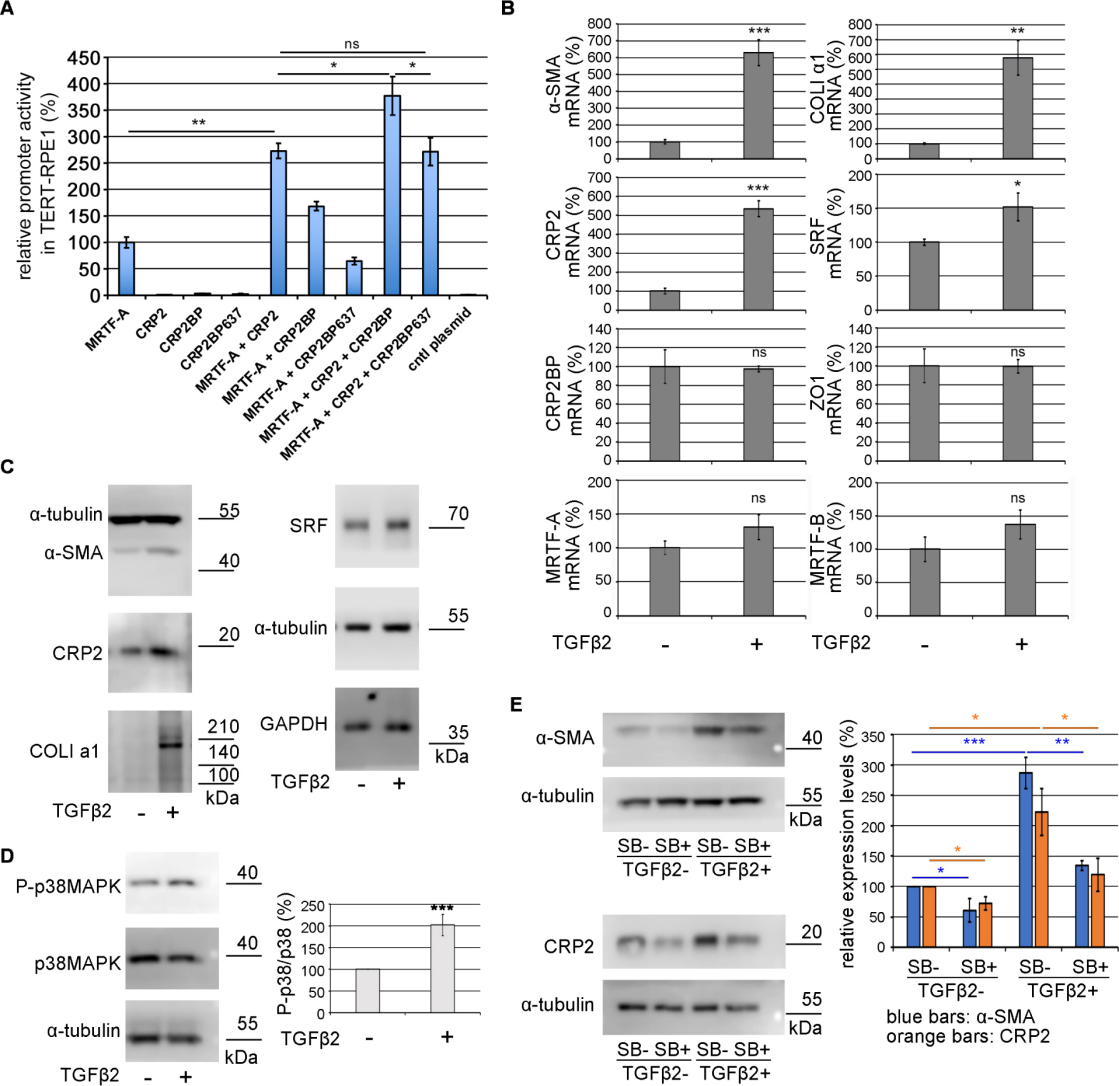
Significance of CRP2 in TGF-β2-induced phenotype transition of hTERT-RPE1 cells (A) Assessment of synergistic effects of MRTF-A, CRP2, and CRP2BP or CRP2BP637 on the CArG-box-dependent promoter activity in hTETR-RPE1 cells. Cells were transfected with 3xCArG-box-Luciferase reporter plasmid, pSVβ-gal, and the indicated plasmids or control plasmid. The promoter activity induced by exogenous MRTF-A alone was set at 100% (means ± SEMs of the results from multiple independent experiments, n = 3). ANOVA shows a significant difference in the promoter activity among the ten different assays (P<0.0001). (B) Growing hTERT-RPE1 cells (70~80% confluent sate) were treated with either vehicle (PBS) containing 0.3% BSA (TGF-β2–) or TGF-β2 (5 ng/ml) for 1 day as described in Materials and Methods. RT-qPCR quantified the expression levels of the mRNAs for myofibroblast markers and related factors. Their levels in non-stimulated cells (TGF-β2–) were set at 100% (means ± SEMs of the results from multiple independent experiments, n = 3). (C) IB analysis confirms the up-regulation of the proteins whose mRNAs increase by TGF-β2 stimulation. Whole-cell lysates from hTERT-RPE1 cells cultured under the indicated conditions were subjected to IB. α-tubulin and GAPDH were used as loading controls. These are representative images from several examinations. (D) Activation of p38MAPK in TGF-β2-stimulated hTERT-RPE1 cells. IB analysis with whole-cell lysates from the indicated hTERT-RPE1 cells (TGF-β2– and TGF-β2+). The graph shows the phosphorylation ratios of p38MAPK (P-p38MAPK/p38MAPK) in the respective cells; the phosphorylation ratio in the control cells (TGF-β2–) was set at 100%. Each value represents the means ± SEMs of the results from multiple independent experiments (n = 3). (E) Effect of the p38MAPK inhibitor on the expression of α-SMA and CRP2. hTERT-RPE1 cells cultured in the presence of either vehicle (DMSO) [SB–] or 10 μM SB20350 (SB) [SB+] for 1 hour were stimulated with vehicle or TGF-β2 for 1 day as described above. Whole-cell lysates from the respective cells were subjected to IB with the indicated antibodies. The graph shows the quantification of the expression of α-SMA and CRP2 proteins. The expression levels of both proteins in the control cells (SB– and TGF-β2–) were set at 100% (means ± SEMs of the results from multiple independent experiments, n = 3). ANOVA shows a significant difference in the expression among the different cultures (P<0.0001 for α-SMA and P = 0.0014 for CRP2). Asterisks indicate the statistical significance of multiple comparisons between the indicated pairs (ns: P>0.05; *: P<0.05; **: P<0.01; ***: P<0.001).

To know whether CRP2 and CRP2BP activate MRTF/SRF/CArG-box-dependent transcription in epithelial cells, we performed the promoter assay in hTERT-RPE1 cells (Fig. 5A). Like in HSFs (Fig. 3B), CRP2 and CRP2BP synergistically activate the promoter activity mediated by MRTF/SRF/CArG-box, suggesting that CRP2 plays a role in a phenotypic transition from epithelial cells to myofibroblasts. We further analyzed the gene expression closely related to this phenotypic transition. Although TGF-β2 stimulation causes a marked increase in the expression of α-SMA, CRP2, and COLI a1, it weakly or less significantly affects the expression of others we examined (Fig. 5B). Fig. 5C shows the up-regulation of α-SMA, CRP2, and COLI a1 at protein levels. Like in HSFs (Hayashi *et al.*, 2023), TGF-β2 stimulation does not induce the expression of CRP2BP in hTERT-RPE1 cells. We then investigated the role of p38MAPK in expression of α-SMA and CRP2. Fig. 5D shows the activation of p38MAPK in hTERT-RPE1 cells stimulated with TGF-β2. Inhibition of this pathway by SB203580 reduces the expression of α-SMA and CRP2 in unstimulated cells and TGF-β2-stimulated cells, respectively (Fig. 5E). These results suggest that, like HSFs, the p38MAPK pathway is critical for the myofibroblastic phenotypic transition of hTERT-RPE1 cells.

**Fig. 6:**
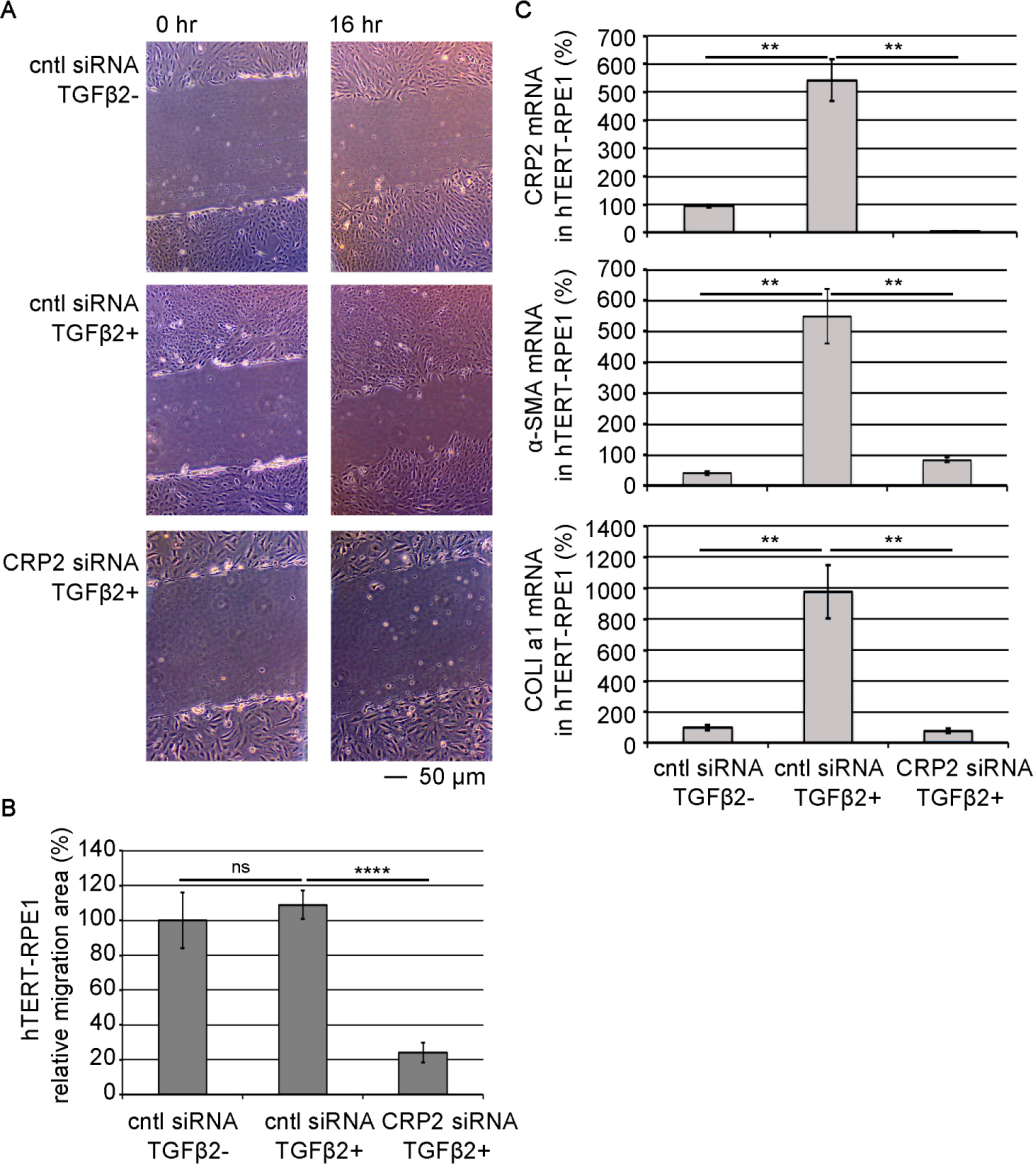
Significance of CRP2 in the cell motility and SMC gene expression in TGF-β2-stimulated hTERT-RPE1 cells hTERT-RPE1 cells were transfected with each siRNAs. One day after siRNA transfection, cells were stimulated with TGF-β2, as described in Materials and Methods. (A and B) Effect of KD of CRP2 on the motility of TGF-β2-stimulated hTERT-RPE1 cells. The cell motility was analyzed by wound healing assay as described in Materials and Methods. Representative images show cells immediately after scratching (0 hours) and at 16 hours (A). The motility of each cell was quantified (means ± SEMs of the results from multiple independent experiments, n = 3) (B). ANOVA shows a significant difference in the cell motility among three different culture conditions (P<0.0001). (C) RT-qPCR quantified the expression levels of the indicated mRNAs. The expression levels in control cells (cntl siRNA transfected cells without TGF-β2) were set at 100% (means ± SEMs of the results from multiple independent experiments, n = 3). ANOVA shows a significant difference in the expression levels of CRP2, α-SMA, and COLI a1 (P<0.0001 for CRP2, P = 0.0010 for α-SMA, P = 0.0002 for COLI a1). Asterisks indicate the statistical significance of multiple comparisons between the indicated pairs (ns: P>0.05; **: P<0.01; ****: P<0.0001).

To examine the role of CRP2 in the myofibroblastic features of TGF-β2-stimulated hTERT-RPE1 cells, we knock-downed the expression of CRP2 by siRNA. TGF-β2 stimulation hardly enhances cell motility, but siRNA-mediated KD of CRP2 reduces cell motility (Fig. 6A, B). The KD of CRP2 also decreases the expression of α-SMA and COLI a1 genes (Fig. 6C). The EMT of RPEs is the cause of retinal fibrosis occurring in developing nAMD (Shu *et al.*, 2020). Aging-induced retinal inflammation causes such phenotypic transition. Myofibroblasts transited from RPEs grow and migrate into the subretinal space and excessively synthesize ECM, resulting in subretinal fibrosis. Our findings suggest that CRP2 may be a novel therapeutic target for retinal degenerative diseases because the KD of CRP2 reduces the myofibroblastic features of hTERT-RPE1 cells induced by TGF-β2 stimulation.

**Fig. 7:**
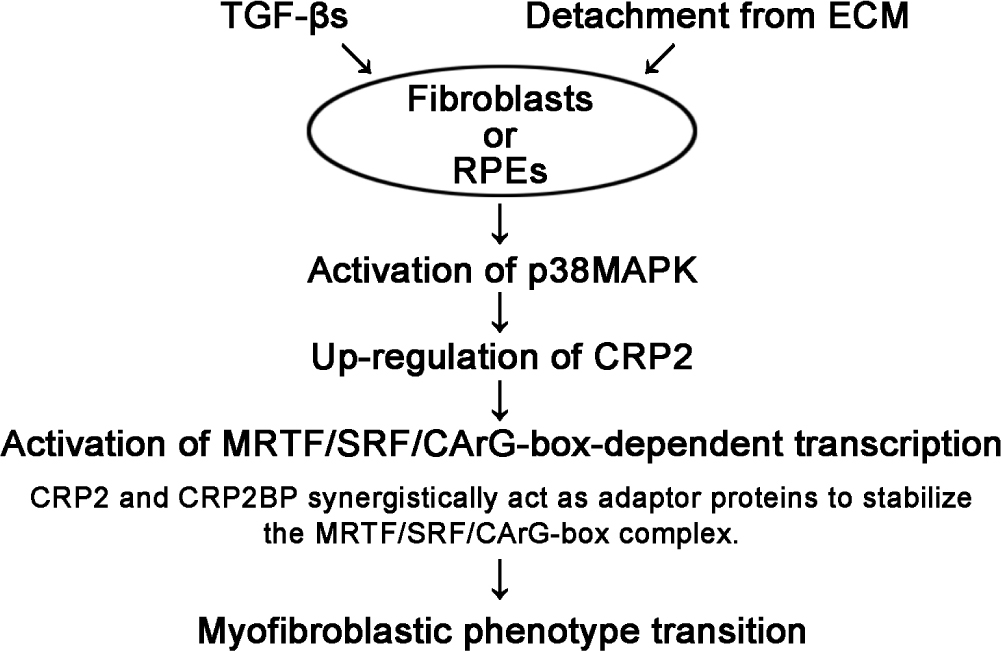
Conclusion remarks Activation of the p38MAPK pathway is critically important for the phenotypic transition from HSFs or RPEs to myofibroblasts. CRP2, whose expression depends on the activation of p38MAPK, activates the transcription mediated by MRTF/SRF/CArG-box followed by the induction of SMC gene expression, leading to the above-mentioned phenotypic transition. In this case, CRP2 acts as an adapter protein to stabilize the complex formed by SRF, MRTF, and CArG-box. Although CRP2BP is known as an epigenetic factor, CRP2BP also acts as an adaptor protein to promote the function of CRP2 mentioned above. Furthermore, cell adhesion to ECM suppresses the phenotypic transition to myofibroblasts because cell detachment from ECM induces the activation of p38MAPK.

Fig. 7 summarizes this study. Activation of the p38MAPK pathway is critically important for the phenotypic transition from HSFs or RPEs to myofibroblasts because p38MAPK plays a role in the expression of CRP2. CRP2 activates the transcription mediated by MRTF/SRF/CArG-box followed by the induction of SMC gene expression, leading to the above-mentioned phenotypic transition. The molecular mechanism is as follows: CRP2 acts as an adapter protein that stabilizes the complex formed by SRF, MRTF, and CArG-box. Although CRP2BP is known as an epigenetic factor, CRP2BP also acts as an adaptor protein to promote the function of CRP2 mentioned above. This CRP2BP ability does not depend on its histone acetyltransferase activity. Furthermore, cell adhesion to ECM suppresses the phenotypic transition to myofibroblasts because cell detachment from ECM induces the activation of p38MAPK.

## Data Availability

All data are contained within this manuscript.

## Author Contributions

K. Hayashi designed the study and performed the experiments with help from T. Morita and R. J. Labios. A. Ashimori, R. Aoki, M. Mikuni, and K. Kimura supervised this project concerning the analysis in hTERT-RPE1 cells. K. Hayashi wrote the manuscript. All authors reviewed and approved the final version of the manuscript.

## Competing Interests

The authors declare no competing financial interests.

## Abbreviations

α-SMA: α-smooth muscle actin
COLI: collagen type I
cntl: control
CRP: cysteine-and glycine-rich protein
CRP2BP: CSRP2-binding protein
ECM: extracellular matrix
EMT: epithelial-mesenchymal transition
GAPDH: glyceraldehyde-3-phosphate dehydrogenase
HA: haemagglutinin
HAT: histone acetyltransferase
HSF: human skin fibroblast
hTERT: human telomerase reverse transcriptase
IB: immunoblot
IP: immunoprecipitation
KD: knock-down
KO: knock-out
MRTF: myocardin-related transcription factor
nAMD: neovascular age-related macular degeneration
ns: not significant
PVDF: poly vinylidene fluoride
RPEs: retinal pigment epithelial cells
SMC: smooth muscle cell
SRF: serum response factor
TAZ: transcriptional co-activator with a PDZ-binding motif
TGF-β: transforming growth factor-β
TGF-βR: transforming growth factor-β receptor
