# Supplementary material for: Significance of the p38MAPK-CRP2 axis in myofibroblastic phenotypic transition
Source: Cell Struct Funct. 2023 Oct 27;48(2):199–210. doi: 10.1247/csf.23060 (PMC11496777; doi:10.1247/csf.23060)
Supplement: Supplementary file 1 — Supplementary Figures [file csf_48_23060_1.zip › 48_23060_1.docx]

**Supplemental Data**

Supplemental Figures (S1-S2)

**
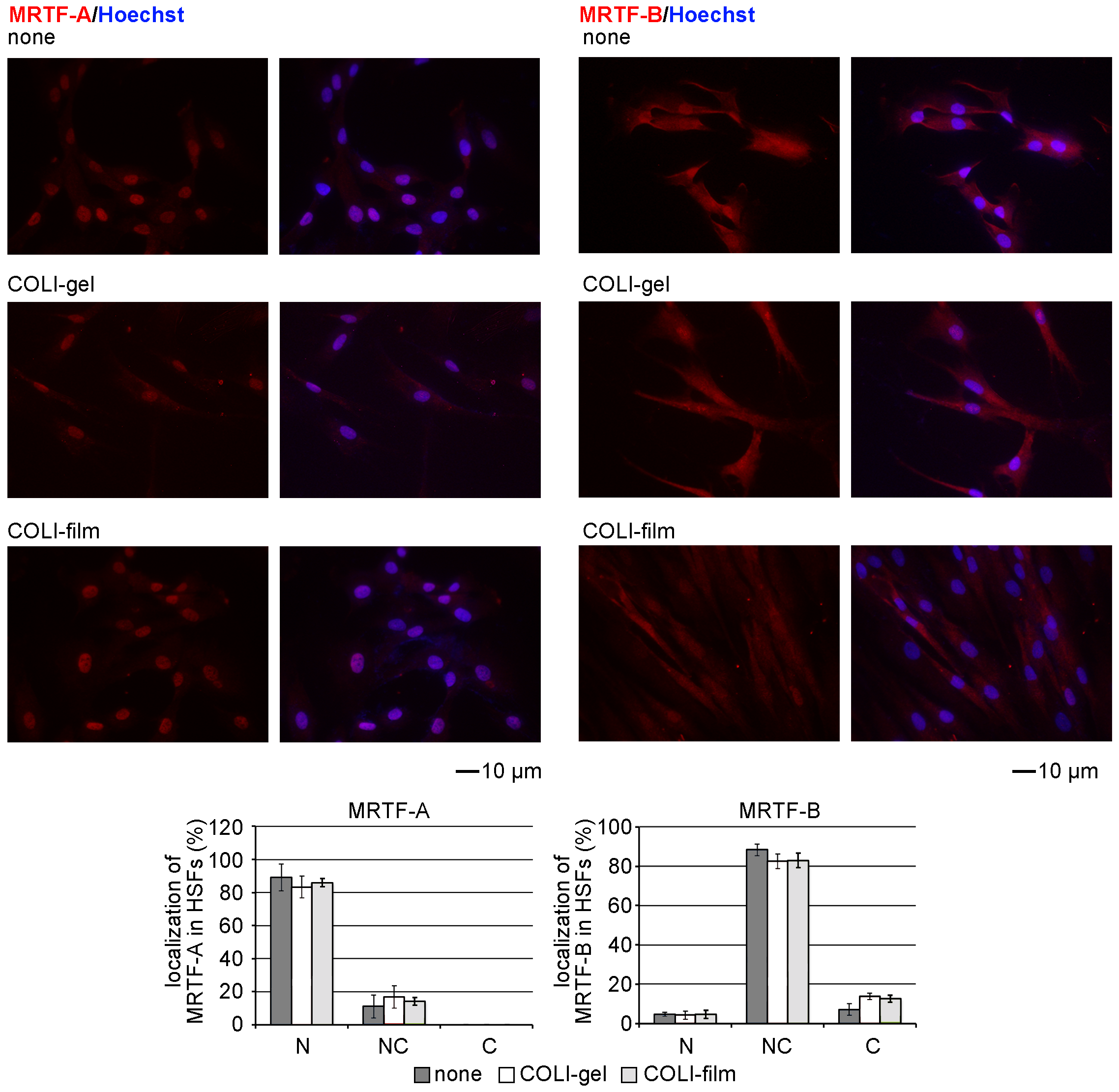
**

**Figure S1. Subcellular localization of MRTF-A and MRTF-B in HSFs cultured on non-coated dishes, COLI-gel, and COLI-film.** Cells were stained with anti-MRTF-A or anti-MRTF-B antibodies and Hoechst 33258. The lower graphs show the quantification of their subcellular localization (means ± SEMs of the results from multiple independent experiments, n = 3). Images were analyzed as described in Materials and Methods: nuclear-specific localization (N), diffuse distribution in the nucleus and the cytoplasm (NC), and cytoplasmic localization (C). ANOVA shows no significant difference in the subcellular localization of MRTF-A or MRTF-B among the three conditions (none, COLI-gel, and COLI-film): P = 1.000 for all combinations among these three. However, MRTF-A and MRTF-B are absolutely in the nucleus and the cytoplasm and nucleus, respectively. Multiple comparisons of the subcellular localization of MRTF-A and MRTF-B are as follows: pairs N-C and N-NC, P < 0.0001 for MRTF-A; pairs N-NC and NC-C, P <0.0001 for MRTF-B.


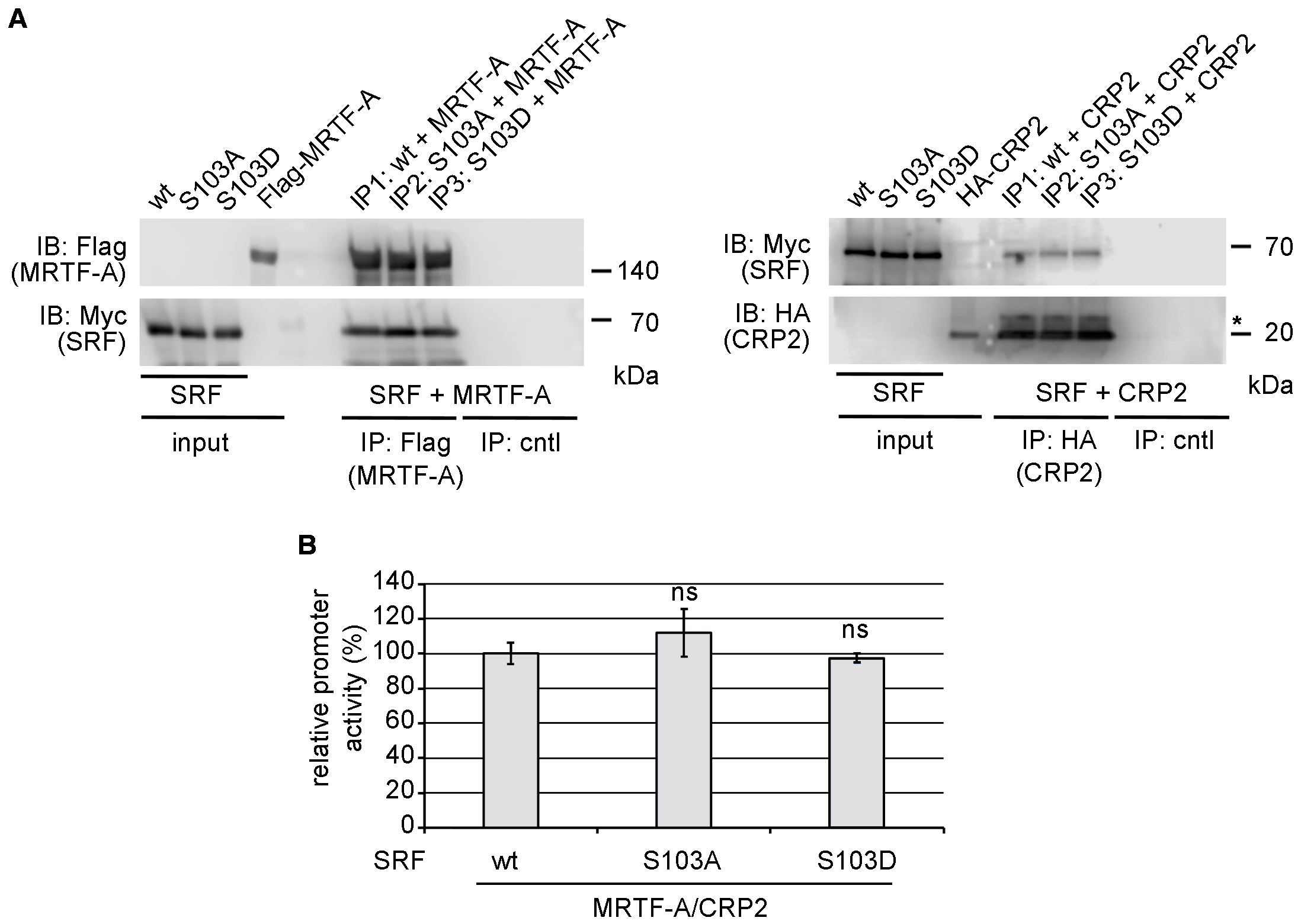


**Figure S2. Effect of SRF phosphorylation by p38MAPK on the association with MRTF-A or CRP2. (A)** Examination of the binding affinity of each mutant SRF (S103D and S103A) to MRTF-A (left panel) and CRP2 (right panel). Protein binding assays were performed using the indicated in vitro translated tagged proteins as described in Materials and Methods. In brief, mixtures of the indicated tagged proteins were immunoprecipitated with a control gel (cntl), anti-FLAG M2 gel (Flag), or anti-HA-affinity matrix (HA). IB shows the respective SRF proteins coimmunoprecipitated with Flag-MRTF-A protein (left) or HA-CRP2 protein (right). Asterisk (*) indicates the IgG light chain. **(B)** Promoter assay in HSFs on non-coated dishes. Cells were transfected with 3xCArG-box-Luciferase reporter plasmid, pSVβ-gal, the expression plasmids for MRTF-A and CRP2, and each of the indicated SRF expression plasmid (wild-type, S103A, or S103D). Promoter activity induced by wild-type SRF (wt) was set at 100% (means ± SEMs of the results from multiple independent experiments, n = 3). The effects of these SRF mutants on the CArG-box-dependent promoter activity are less significant (ANOVA P = 0.7176). The multiple comparisons between SRF wt and SRF S103S or between SRF wt and SRF S103D are not significant (ns).
